# Aligned nanofibrous collagen membranes from fish swim bladder as a tough and acid-resistant suture for pH-regulated stomach perforation and tendon rupture

**DOI:** 10.1186/s40824-022-00306-1

**Published:** 2022-11-08

**Authors:** Zhaohui Luan, Shuang Liu, Wei Wang, Kaige Xu, Shaosong Ye, Ruijue Dan, Hong Zhang, Zhenzhen Shu, Tongchuan Wang, Chaoqiang Fan, Malcolm Xing, Shiming Yang

**Affiliations:** 1 Department of Gastroenterology, Xinqiao Hospital, Army Medical University, NO.183, Xinqiao Street, Chongqing, 400037 China; 2 Department of Mechanical Engineering, University of Manitoba, Winnipeg, MB R3T 2N2 Canada; 3 Department of Gastroenterology, Affiliated Hospital of North Sichuan Medical College, Maoyuan Nan Road, Shunqing District, Nanchong City, Sichuan China; 4 Chongqing Municipality Clinical Research Center for Gastroenterology, Chongqing, China

**Keywords:** Aligned collagen fibrous swim bladder, Smart tough suture, Stomach healing, Pig models, Achilles tendon

## Abstract

**Background:**

Wound closure in the complex body environment places higher requirements on suture’s mechanical and biological performance. In the scenario of frequent mechanical gastric motility and extremely low pH, single functional sutures have limitations in dealing with stomach bleeding trauma where the normal healing will get deteriorated in acid. It necessitates to advance suture, which can regulate wounds, resist acid and intelligently sense stomach pH.

**Methods:**

Based on fish swim bladder, a double-stranded drug-loaded suture was fabricated. Its cytotoxicity, histocompatibility, mechanical properties, acid resistance and multiple functions were verified. Also, suture’s performance suturing gastric wounds and Achilles tendon was verified in an in vivo model.

**Results:**

By investigating the swim bladder’s multi-scale structure, the aligned tough collagen fibrous membrane can resist high hydrostatic pressure. We report that the multi-functional sutures on the twisted and aligned collagen fibers have acid resistance and low tissue reaction. Working with an implantable “capsule robot”, the smart suture can inhibit gastric acid secretion, curb the prolonged stomach bleeding and monitor real-time pH changes in rabbits and pigs. The suture can promote stomach healing and is strong enough to stitch the fractured Achilles tendon.

**Conclusions:**

As a drug-loaded absorbable suture, the suture shows excellent performance and good application prospect in clinical work.

**Supplementary Information:**

The online version contains supplementary material available at 10.1186/s40824-022-00306-1.

## Introduction

The suture is purposed to hold tissues together. With an irreplaceable role in operations, it facilitates healing and leaves minimal scar after injury [1, 2]. Despite gratifying tissue adhesives [3–6], sutures still comprise the most considerable portion of the wound closure market with over 5 billion dollars yearly [7, 8]. Most used absorbable sutures are catgut (natural) and polyglycolic-co-lactic acid (PGLA) (synthetic). Catgut’s strength wears off quickly with persistent tissue reaction [9, 10]. Besides their complex fabrication processes, the synthetic absorbable sutures show high friction with induced microtrauma [11]. Those lead to the complications of bleeding, dehiscence and infection [12]. It makes necessary to advance multi-functional sutures in wound management. Antibacterial sutures prevent surgical site infections [11, 13]. Bioactive sutures promote healing [14, 15]. A smart suture can sense wounds’ temperature, pH and bacterial counts [1, 2, 13, 16]. Critically, loading bioactive molecules and smart sensing upon different needs in one suture would be a crucial advancement.

Collagen is the most abundant protein in animals and comprised of a right-handed bundle of three parallel, left-handed polyproline II-type helices [17], possessing characteristics of biodegradable, biocompatible, low antigenic and a certain strength [18, 19]. After crosslinking, stiffness, tensile strength, thermal stability and resistance to enzymatic hydrolysis significantly improve [20–24]. With these characteristics, collagen has been maturely used as suture material in the catgut. Currently, fish-derived collagen has been reported as an effective substitute for mammalian collagen with low risk of pathogen infections and without religious restrictions [25]. Known for its tough in strength, swim bladder (SB) fills and exhausts air with no fatigue-induced damage to have fish floating up and down [26, 27]. It is mainly composed of collagen I, and also contains elastin and glycosaminoglycan. With all these characteristics of collagen and advantage of fish-derived collagen, SB perfectly meets the key parameters such as strength, sterility, absorbability, histocompatibility and multi-function for suture application [2, 28], and is qualified for an ideal absorbable suture material. A large number of swim bladders are wasted every year in the world, and the development of swim bladders into sutures can significantly increase economic value.

**Scheme 1:**
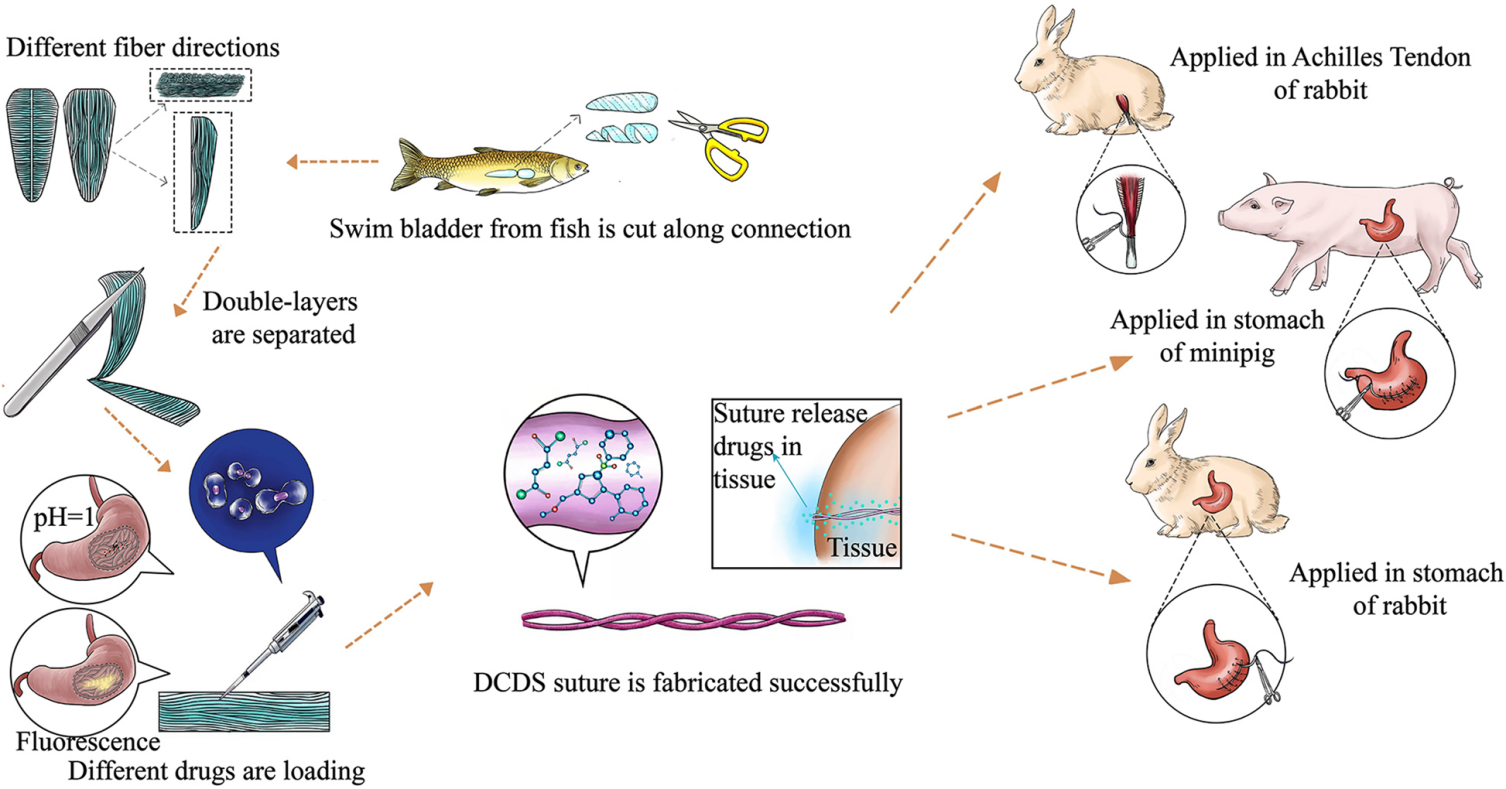
The schematic illustration of multi-functional DCDS suture from fish swim bladder and its application in repairing stomach and tendon

In this work, we report a multi-functional smart suture – decellularized crosslinked double-strand swim bladder (DCDS) suture used in rabbits and minipigs (Scheme 1). We tested tensile strength, foreign body reaction and inflammation reaction, showing obvious advantages over conventional catgut suture. Stomach environment is in an extremely low pH, which is more likely to induce wound bleeding when injury happens [29–31]. In addition, acid corrosion to suture will significantly affect its performance [32]. It causes suture breaking and stomach content flowing into the abdominal cavity, leading to severe peritonitis, even life-threatening. These propose higher requirements for sutures used in gastric surgery. To show the advantages of DCDS suture in the stomach, acid resistance and mechanical property in a stimulated stomach environment were tested. With the characteristic of micropores, DCDS suture can load drugs for controlled releases. Acid-suppressing drug, Vonoprazan Fumarate (VF), was loaded to test the inhibiting effect of gastric acid secretion in the stomach. Furthermore, the suture can be modified to monitor pH in the stomach. Besides, DCDS suture with acidic fibroblast growth factor (AFGF) was tested to promote wound healing. The suture in this work is also systematically compared with other reported sutures with biological origin in Table 1 [2, 8, 33, 34].

**Table 1:** Comparison of DCDS and other sutures with biological origin

Suture	Biological origin	Main ingredient	Tensile strength (Mpa)	Absorbability	Histocompatibility	Characteristics
**DCDS**	Swim bladder from fish	Collagen	442–553	Absorbable	Good, better than gut with very mild tissue inflammation	1. Achieving multi-function with drugs. 2. Obtaining different crosslinking degree to achieve different strengths and degradability on different needs. 3. Possessing strong acid resistance;
**Plain gut**	Gut of mammalian	Collagen	235–413	Absorbable	Causing tissue inflammation in acute phase	Easy to break with rapid loss of strength and rapid degradation in vivo.
**Chromic gut**	Gut of mammalian	Collagen	224–410	Absorbable	Tissue inflammation lighter than plain gut	Comparing with plain gut, the strength maintenance in vivo is improved and degradation is slower after chrome.
**Silk sutures with antibacterial and anti-inflammatory functions**	Silk	Silk fibroin	399–441	Non-absorbable	Causing tissue inflammation in the beginning short term	Based on silk suture, it achieves drug release and promoting wound healing with loading drug.
**Opto-Electro Sensing Sutures**	Silk	Silk protein	Uncertain (Depending on core material)	Uncertain (Depending on core material)	Depending on core material	1. Multilayer drug loading. 2. Silk protein is only used as a coating material, and the property of the suture mostly depends on the inner core material;
**Light-Activated Tissue-Integrating Sutures**	Rat tail tendon	Collagen	slightly lower than PGA	Absorbable	Causing mild tissue inflammation	It can closely combine with the tissue and promote tissue healing with light activation.

## Materials and methods

### Fabrication of DCDS sutures

Decellularized swim bladder: Fresh swim bladders from Grass Carp were collected from the local Farmer’s Market. Swim bladders were cut along the connection to obtain 2 pieces of fibrous tissue membranes. Fat, attached mucous membrane and blood vessels on the surface were removed. Fibrous tissue was treated with 0.1% sodium dodecyl sulfate (SDS) (Sangon Biotech, Shanghai) for 24 hours at room temperature with gentle shaking for 3 times. Then fibrous tissue was treated with DNase/RNase (Aladdin, shanghai) for 2 hours. Finally, PBS solution was used to wash away the residue of different reagents.

Separation of decellularized inner-layer membrane: The decellularized fibrous tissue of the swim bladder was placed on a hard plastic board with inner-layer upward. The two-layer structure was separated to obtain the inner-layer membrane. It was dried in the oven at 30 °C for 5 hours.

Fabrication of decellularized crosslinked single-strand swim bladder (DCSS) suture: The decellularized inner-layer membrane was trimmed into 0.5 cm in width, then was moved onto the hard plastic board with distilled water. The required drug solution or suspension was coated onto the membrane (0.5 cm in width). After 3–5 minutes at room temperature, the membrane was rolled up. A 15 cm long decellularized un-crosslinked single-strand swim bladder (DUSS) suture by onion-like wrapping was obtained. DUSS suture was submerged in the EDC/NHS crosslinking for 4 hours and was washed with distilled water for 2–3 times, each time for 5 minutes. The DCSS suture was then obtained.

Fabrication of DCDS suture: Two DUSS sutures were twisted into a double helix structure to obtain the decellularized un-crosslinked double-strand swim bladder suture with about 4 turns/cm. Then it was submerged in the EDC/NHS crosslinker solution for 4 hours and was washed with distilled water for 2–3 times. The DCDS suture was obtained.

### Fabrication of drug solution or suspension coating with DCDS sutures

VF suspension: 25 mg Vonoprazan Fumarate (TAK-438) (Aladdin, Shanghai) was dissolved as a suspension in 1.25 mL PBS solution (pH = 7.0). The suspension was stored in 4 °C freezer. DCDS suture was laden with 100 μL VF suspension (2 mg VF).

AFGF solution: 10 μg rat AFGF (Novoprotein, Shanghai) was dissolved in 1 mL sterilized double-distilled water to obtain a 10 μg/mL AFGF solution. DCDS suture was coated with 100 μL AFGF solution (1 μg AFGF).

Fluorescein sodium solution: 10 mg Fluorescein sodium salt (Solarbio, Beijing) was dissolved in 1 mL double-distilled water to obtain 10 mg/mL fluorescein sodium solution. DCDS suture was laden with 100 μL fluorescein sodium solution (1 mg fluorescein sodium).

Congo red pH indicator solution: 5 mg Congo red (Sangon Biotech, Shanghai) powder was dissolved in 1 mL 10% ethanol solution to obtain 5 mg/mL Congo red pH indicator solution. DCDS suture was laden with 100 μL Congo red pH indicator solution (0.5 mg Congo red).

### Histology staining

Staining of swim bladder: Hematoxylin-eosin (HE) staining (Biospes, Chongqing) was carried out on swim bladder before and after decellularization to observe distribution of cells and fibers. Masson’s trichrome (Jiancheng, Nanjing) and Sirius red (Biospes, Chongqing) staining were operated to observe collagen. Verhoeff-Van Gieson (Baso, Zhuhai) was operated to detect elastin’s existence, and Safranine O (Solarbio, Beijing) for glycosaminoglycan.

Fast freezing section: 1 cm length of DCSS and DCDS sutures froze in an optimal cutting temperature compound and were cut into 8 μm thickness with microtome (Leica CM1860, German) to observe the cross-section and long-axis.

Staining of animal stomach specimens: With endoscopy, tissues were clamped from the sutured wound of the rabbit stomach after 3 days, 7 days, 2 weeks and 4 weeks. Tissues were clamped from the sutured wound of minipig stomach with endoscopy after 7 days. After washed with PBS solution, the collected tissues were immediately fixed in 4% paraformaldehyde. Four weeks after surgery of gastric fistula, rabbits and minipigs were euthanized, and tissues around sutured wound were collected and also fixed in 4% paraformaldehyde after washing. After embedded into paraffin wax, all samples were sectioned at a thickness of 2.5 μm with the microtome (Leica RM2255, German) for HE staining and immunostaining analysis. CD31 (Affinity, America), α-Smooth muscle actin (α-SMA) (Sanying, Wuhan) and Proliferating cell nuclear antigen (PCNA) (Sangon Biotech, Shanghai) were carried out to observe the proliferation and healing of the wound.

The stained sections were scanned by digital pathological section scanner, read and screenshotted by software of Image Scope, and 3–4 fields of view in each sample were used.

### Cell experiment

Cytotoxicity test: DCDS suture was submerged in a medium composed of 90% DMEM and 10% Fetal Bovine Serum for 24 hours, and the medium was used to culture cells as the experimental group. Pure medium without submerging DCDS suture was used as the control group. 3T3 cells were cultured with the medium of the experimental and control group. After 1, 2 and 3 days, the number of cells in two groups was detected with a micro plate spectrophotometer. Statistical analysis of Independent-Samples t Test was operated to compare the difference of the two groups. Cell colonization test: 3T3 cells were put into the medium for co-cultivation with decellularized crosslinked membrane (DCM) for 2 days. DCM was taken out and washed with PBS solution (pH = 7.0) to wash away un-colonized cells. Then DCM was observed directly and after staining with cell viability assay kit* green/red dual fluorescence (Sangon Biotech, Shanghai) under a fluorescence microscope (Olympus, Japan). It was detected whether cells could colonize on the surface of DCM.

### Scanning Electron microscope (SEM) characterization

The microstructure of the swim bladder membrane and suture was observed with SEM (Crossbeam 340, Zeiss). Decellularized un-crosslinked membrane (DUM) and DCM were dried in an oven at 30 °C for 5 hours and trimmed into 5 ✕ 5 mm. DCSS, DCDS and catgut sutures were dried in an oven at 30 °C for 5 hours. They were cut, frozen with liquid nitrogen and stretched to break, and trimmed into 5 mm length to observe the fracture ends. DUM (5 ✕ 5 mm) and DCM (5 ✕ 5 mm) with outer side, inner side and cross-section upward, and DCSS, DCDS, and catgut sutures with cross-section and long-axis upward were conductive-coated with gold.

### Spectroscopy characterization

Fourier transform infrared (FTIR) spectra of un-crosslinked and crosslinked swim bladder were recorded on a Perkin-Elmer FTIR spectrometer (Spectrum100, PerkinElmer). DUM (5 ✕ 5 mm) and DCM (5 ✕ 5 mm) were tested under Raman spectrometer.

### Mechanical test

DCSS, DCDS and catgut sutures in the length of 6 cm were prepared. Diameters were measured at the top, middle and bottom with a vernier calliper and the average diameter was calculated. Sutures were fixed between clamps, and tensile strength was measured in dry (in the oven at 30 °C for 5 hours) and wet (soaking in PBS solution for 30 min) state with universal material testing machine (Sanfeng, Jiangsu). Sutures were measured with the initial gauge length 25 mm, at the speed of 10 mm/min. Inner-layer DUM and DCM (IDUM/IDCM) were trimmed into 1 ✕ 6 cm, and the average thickness was obtained. Wet strength was measured with universal material testing machine as above. For the fracture energy of sutures, the energy was needed by the unit area of the suture from beginning to failure (Stress: N/m^2^, Displacement: m) [35]. Young’s modulus was obtained after measurement.

### Acid resistance test

Anti-acid test: DCSS and catgut sutures in 6 cm length were completely submerged into hydrochloric acid solution (pH = 1.0). After different days (1, 2, 3, 5 and 7 days), sutures were taken out and washed with PBS solution (pH = 7.0) for 5 minutes for two times. Then sutures were measured with universal material testing machine for wet strength and observed under SEM.

Mass loss rate: DCDS and catgut sutures in the length of 3–4 cm were submerged in PBS solution (pH = 7.0) for 1 day. They were taken out and put in an oven for drying under 30 °C for 5 hours and were weighed (m_1_). Then they were submerged in hydrochloric acid solution (pH = 1.0) for 1, 2, 3, 5 and 7 days, respectively, and washed with PBS solution (pH = 7.0) and then moved to the oven at 30 °C for 5 hours again before weighed again (m_2_). The mass loss rate (M) was calculated: M = (m1-m2)/m1.

### Crystallinity detection

DCDS and catgut sutures were tested with small/wide angle X-ray scattering (SAXS/WAXS) (Xenocs, France) to performed X-ray diffraction (XRD).

### Drug release

Fluorescein sodium loaded DCDS: DCDS suture with 1 mg fluorescein sodium was submerged in 20 mL PBS solution (pH = 7.0). 100 μL solution was pipetted after 10 minutes, 30 minutes, 1, 2, 6, 12, 24, 48 and 72 hours. Then concentrations of fluorescein sodium at different time points were detected with a microplate spectrophotometer.

VF loaded DCDS: DCDS suture with 2 mg VF was submerged in 20 mL PBS solution (pH = 7.0) and pipetted at the same time as for fluorescein sodium. The concentration was detected with ultra-performance liquid chromatography (UPLC) (Walers, Singapore).

### pH monitoring

In vitro: DCSS sutures with Congo red were submerged respectively in PBS solutions of pH = 1, 4 and 7, and then were taken out for color comparing. Image J software was used to analyze the grayscale. DCSS sutures with Congo red were submerged in PBS solution of pH = 1, then to pH = 7 for 3 repeats. Color transition was observed during this process.

In vivo: DCDS suture (one strand with Congo red, the other with VF) was used to stitch gastric fistula in adult male rabbit (2.5 Kg). The color was observed after 12 and 36 hours with gastroscopy.

### In vivo assay

All animal work was approved by the Laboratory Animal Welfare and Ethics Committee of the Third Military Medical University (AMUWEC20202192). Rats used in this work were juvenile male rats from Dashuo Experimental Animal Co., Ltd. Rabbits were adult male rabbits from Yuda Rabbit Farm. Minipigs were juvenile male minipigs from Yingeng Farm.

Subcutaneous implantation for degradation: IDCM was cut into 5 ✕ 5 mm size and the catgut suture was cut into 1 cm in length. IDCM and segmental catgut sutures were implanted subcutaneously in rats (45–65 g) after sterilization with ultraviolet (UV). The tissue samples with implants were taken for observation and SEM investigation after 2, 4, 8 and 16 weeks. They were also for HE staining after 1 day, 7 days, 4 weeks and 8 weeks.

Gastric fistula suture in rabbits: The wound with 0.7–0.8 cm diameter was made as a fistula. The mucosal layer and then the serosal layer were sutured with DCDS and catgut sutures. The endoscopic observation was performed after 3 days, 7 days, 2 weeks and 4 weeks with biopsy around the wound. Rabbits were euthanized after 4 weeks, and stomach tissue of the wound was obtained. Tissues were for HE and immunohistochemical staining.

Gastric fistula suture in minipigs with pH monitoring: Minipigs (15–20 Kg) were used for this study. The process of surgery was same as rabbit with DCDS suture with VF and AFGF. Then pH wireless meter (Jinshan, Chongqing) was calibrated, sent into the stomach and fixed by titanium clamps around the wound to monitor pH for 4 days. After 7 days, minipigs were operated with “capsule robot” to observe wound healing, and a biopsy was taken. After 4 weeks, minipigs were again observed and then were euthanized to obtain stomach tissue of wound for HE and immunohistochemical staining.

Achilles tendon suture: Rabbits (2–2.5 Kg) were used for this study. After the Achilles tendon was exposed and completely cut off, the Kessler method was used to suture the ruptured Achilles tendon. The ruptured Achilles tendon was not sutured in the control group. The wound was bandaged and the left leg (operation side) was fixed with a plaster for 5 days. Rabbits’ movement was observed after 7 and 14 days. The operated Achilles tendon was removed for the stretch test (1 day and 1 month) and HE staining to evaluate the healing.

### Statistical analysis

All data were presented as Mean ± SD to evaluate the differences between the values (Independent Samples t-Test). *P* < 0.05 was considered statistically significant.

## Results

### Structure and composition of SB

**Fig. 1:**
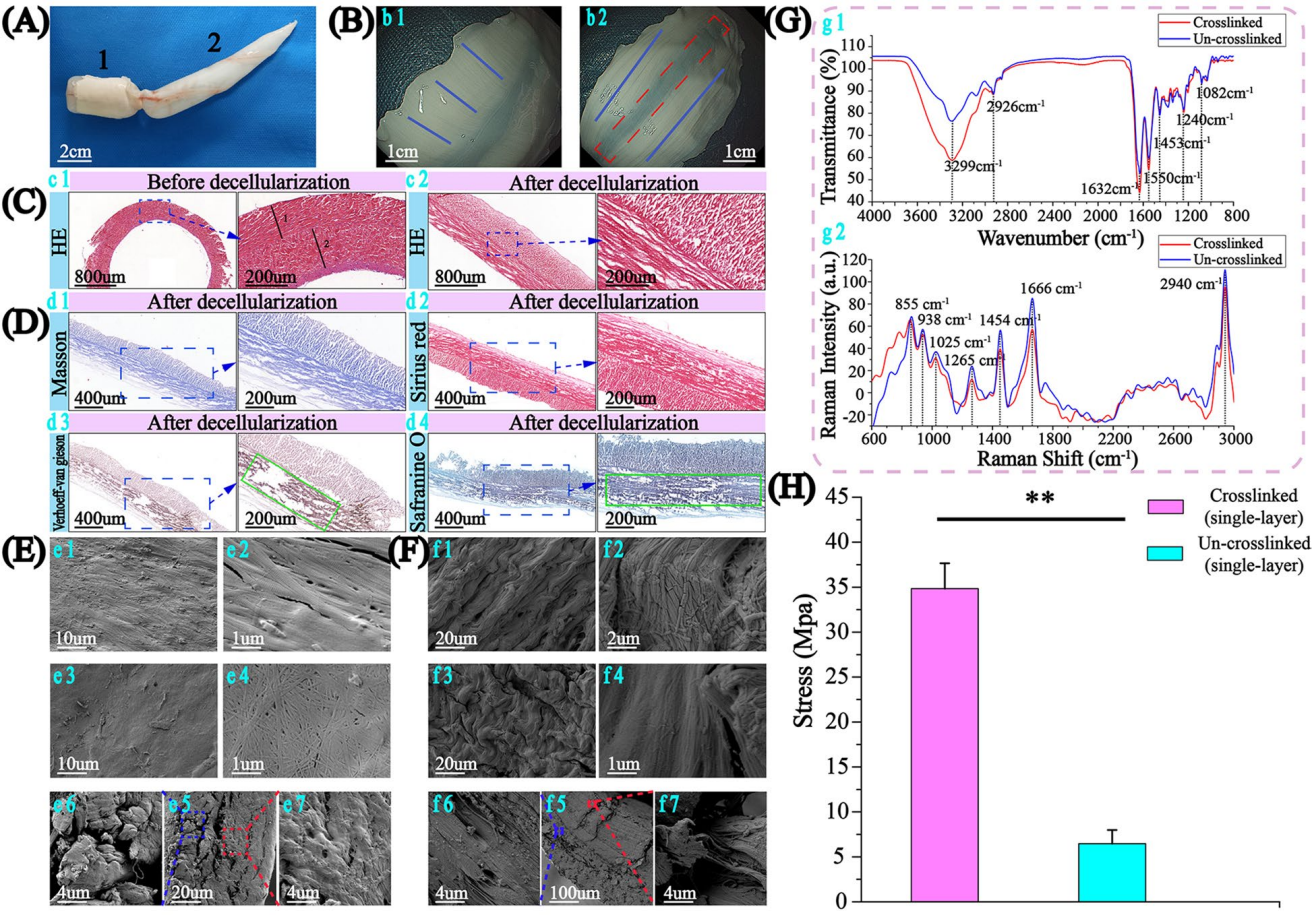
The morphology and structure and mechanical properties of SB. **A** A complete SB of grass carp with two chambers (1: stubby cylinder, 2: slender cone). **B** Fiber orientation of the outer layer (b1- blue line) and the inner layer (b2- blue line) are vertical. Red dotted frame (b2) showing the connecting part, which connects 2 pieces of fibrous tissue spirally to form SB. **C** SB HE staining for primary (c1) and decellularized (c2). **D** Decellularized SB cross-section by staining: Masson (d1), Sirius red (d2), Verhoeff-Van Gieson (d3) and Safranine O (d4). **E** SEM of inner layer (e1, e2), outer layer (e3, e4), and their cross-section (e5–7) of DUM. **F** SEM of inner layer (f1, f2), outer layer (f3, f4) and cross-section (f5–7) of DCM. **G** The Infrared spectroscopy (g1) and Raman spectroscopy (g2) of DUM and DCM. **H** The wet strength of IDUM and IDCM (*n* = 3)

**Fig. 2:**
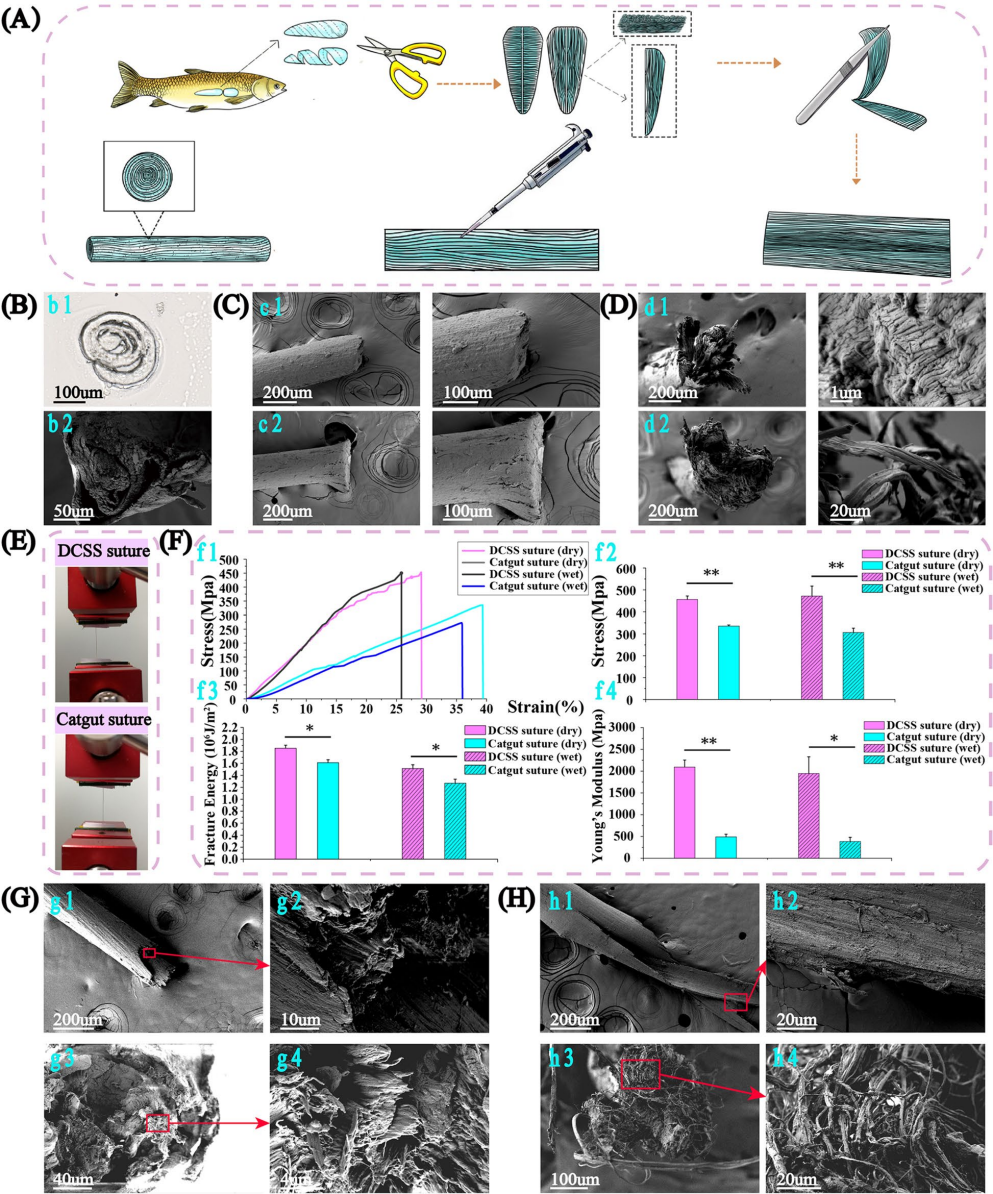
DCSS suture fabrication, morphology at the cross-sections, and morphology at breakage interface and tensile strength. **A** Fabrication of DCSS suture on decellularization, inner layer separation, drug loading and rolling. **B** The frozen section (b1) and SEM images (b2) of DCSS suture. Longitude (**C**) and cross-section (**D**) of two types of sutures under SEM (c1: longitude of DCSS suture after cutting off using scissor, c2: longitude of catgut suture after cutting, d1: breakage interface of DCSS suture after freezing, d2: breakage interface of catgut suture after freezing). **E**, **F** DCSS and catgut sutures’ tensile strength, fracture energy and Young’s modulus in the dry and wet state (*n* = 3). After stretching, the fracture interface of DCSS (**G**) and catgut suture (**H**)

SB used in this work was from grass carp, containing 2 chambers. The large chamber was in the shape of stubby cylinder (Fig. 1A,1), and the small was a slender cone (Fig. 1A,2), communicating with each other through a central tube. Composed of fibrous tissue and connecting part (red frame), a small chamber was selected for suture fabrication because of longer fiber (In the following, “SB” refers to the membrane from the small chamber).

Fibers in outer surface of membrane were short (Fig. 1b1), perpendicular to inner surface where fibers were longer (Fig. 1b2). HE staining showed fibers were divided into 2 layers (Fig. 1c1, 1 and 2). Fibers are closely clustered and aligned in the same layer. Fibers of different layers are perpendicular to each other (Fig. 1c1). After decellularization, fibers became loose with structure in pristine without damage (Fig. 1c2).

Blue in Masson and red in Sirius red staining showed collagen as major composition. Black in Verhoeff-Van Gieson and Safranine O staining (green square) respectively suggested elastin and glycosaminoglycan (Fig. 1D). Compared with the DUM, from the SEM, fiber morphology in the DCM changed. Fibers in DUM were loose and less directional, especially in the outer layer (Fig. 1e1–4). But they were in bundles with the same direction in DCM, just like reinforced cement structure. They were tighter, more directional and denser than DUM (Fig. 1f1–4). Two-layer structure was also observed with more closed fibers in DCM than DUM (Fig. 1e5–7, f5–7). For longer length, better directionality and density, inner layer was chosen to fabricate suture.

From infrared spectroscopy of DUM and DCM, transmittance of DCM at 1240 cm^− 1^ (Amide III), 1550 cm^− 1^ (Amide II), 1632 cm^− 1^ (Amide I) and 3299 cm^− 1^ (Amide A) [36–40] were lower, indicating amide in DCM was more, consistent with crosslinking process of forming amide (Fig. 1g1). Raman peaks at 855 cm^− 1^ (collagen), 938 cm^− 1^ (ν(C-C) skeletal of collagen backbone), 1025 cm^− 1^ (carbohydrates) and 1265 cm^− 1^ (Amide III) [41–47] showed collagen (Fig. 1g2). Peaks at 1454 cm^− 1^ (CH3 bending and CH2 scissoring associated with elastin) [48] and 1668 cm^− 1^ (Amide I) [39] were consistent with elastin. DUM and DCM were similar, suggesting that the composition didn’t change significantly after crosslinking. IDUM and IDCM were tested in mechanics (Fig. 1H). After crosslinking, the wet strength of IDCM rose to 6 folds, and fibers were closed packed and more directional, effectively preventing small molecules invading. This may explain why the wet strength of IDCM is higher.

### Fabrication, morphology and tensile strength of the DCSS sutures

DCSS suture was obtained on decellularization, inner layer separation, drug loading, rolling and crosslinking (Fig. 2A, Video 1, Supplementary Fig.S3). Its cross-section was composed of concentric circles from inside to outside under frozen section staining and SEM, which was onion-like wrapped (Fig. 2b1–2). Compared with the loose, cracked section of catgut, DCSS suture demonstrated no visible deformation and good integrity after cutting (Fig. 2C), showing excellent ability to resist deformation and shearing force.

Different from completely randomly distributed fibers in catgut, DCSS sutures still maintained better integrity dense and consistent fiber arrangement after frozen (Fig. 2D). This well responded to the following mechanical properties (Fig. 2F). The maximum strain of DCSS suture in dry (28%) was lower than catgut (39%), but stress (450Mpa) was much higher than catgut (340Mpa). In wet, stress didn’t reduce significantly in DCSS suture, while it decreased about 50Mpa in catgut. Fracture energy of DCSS suture was higher than catgut in both dry and wet, with a significant difference, suggesting that it can withstand more energy before breakage. Young’s modulus of DCSS suture was also higher, indicating DCSS suture can better resist shape changes. With higher strength, fracture energy and rigidity, DCSS suture can maintain more durable and stable in strength to bridge wound together even within body fluids to avoid failure.

After stretching, the fracture interface of DCSS suture (Fig. 2g1–2) was more uniform than the irregular catgut (Fig. 2h1–2). In cross-section, fibers were clustered in bundles with a high orientation for DCSS suture (Fig. 2g3–4), while very random in catgut, without order or directionality (Fig. 2h3–4). The crosslinking improved fibers bonding and maintained good directionality which contributed to the resisting under exterior stretching. In addition to collagen, DCSS also contained elastin which increased the elasticity to withstand higher energy before breaking [49].

### Decellularized crosslinked double-strand SB (DCDS) suture

**Fig. 3:**
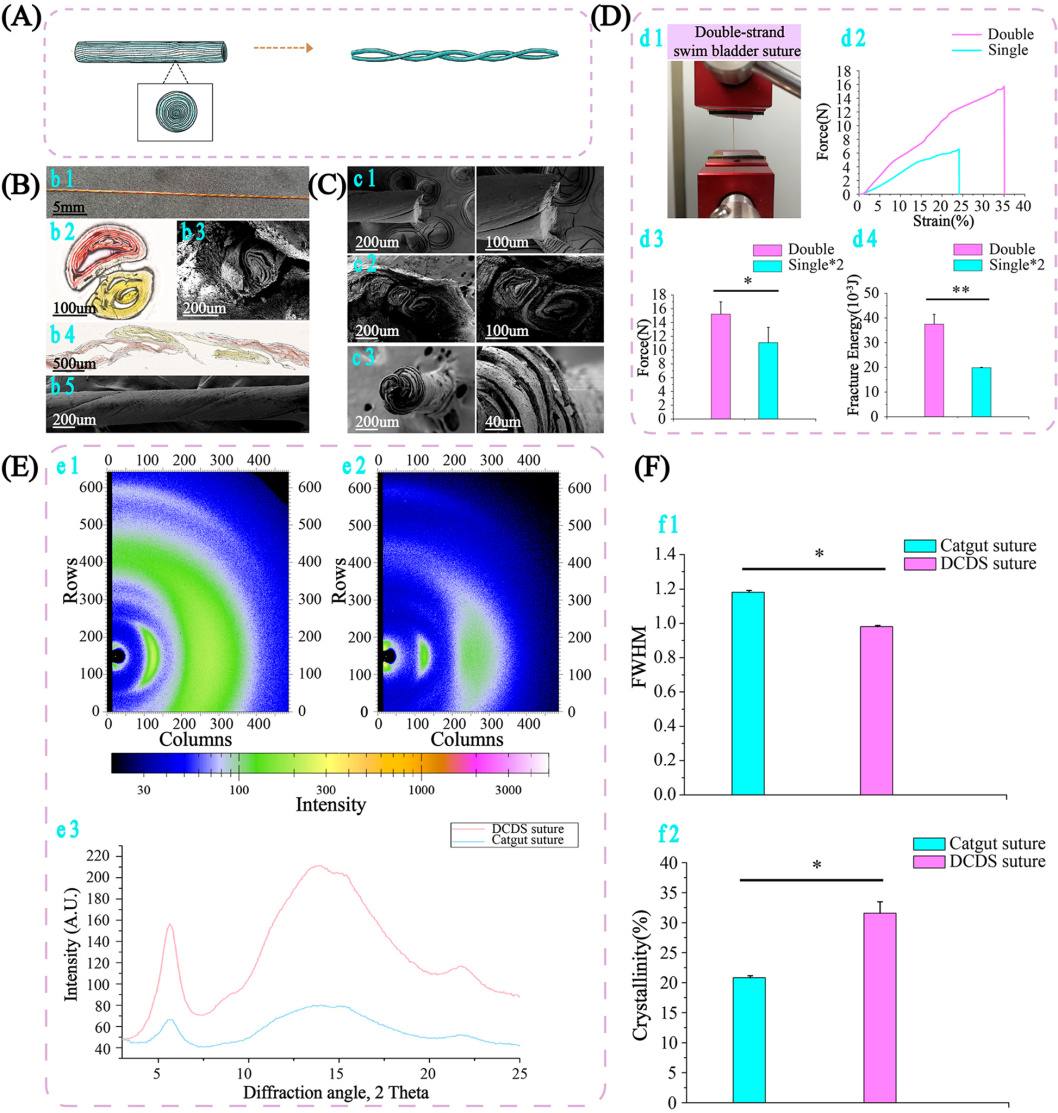
Fabrication of DCDS suture, morphology, tensile strength and structural state during stretching. **A** Fabrication of DCDS suture via DCSS. **B** DCDS suture laden with fluorescein sodium and methyl orange (b1) showing the double-strand state. The frozen staining and SEM of DCDS suture (b2: frozen section image, b3: SEM image, b4: frozen section in longitude, b5: SEM image in longitude). **C** Longitude and cross-section of DCDS suture under SEM (c1: longitude, c2: cut end after stitching in tissue, c3: broken end after freezing). **D** Tensile strength and fracture energy of DCSS (single) and DCDS (double) suture after in PBS for 10 minutes (*n* = 3). **E** X-ray diffraction of DCDS (e1), catgut suture (e2) and graph with peaks (e3). **F** Histogram of FWHM and crystallinity of two sutures (*n* = 3)

Multi-filament structured sutures improve mechanical property than monofilament [50]. Therefore, based on DCSS (single strand), DCDS (double strand) suture was fabricated (Fig. 3A). Two DCSS filaments, each loaded with fluorescein sodium and methyl orange, were woven spirally to form a DCDS suture (Fig. 3B). Frozen staining and SEM showed two concentric semicircles with the rolling trace from inside to outside closely fit together in cross-section, and two single-strand interlaced with spiral weaving state in long-axis section. The cut end of DCDS suture was neat with no visible deformation and damage (Fig. 3c1), which was similar to DCSS. After suturing in tissue for 2 weeks, DCDS suture fit closely with surrounding tissue, showing good interaction with tissue (Fig. 3c2). Frozen cross-section showed the broken fiber bundles orderly distributed along the spiral direction (Fig. 3c3). It was in much more order than catgut. In the wet state, DCDS suture’s maximum strain (35%) was significantly higher than DCSS (24%). Its breaking force (16 N) was higher than 2 folds of DCSS, and so was fracture energy (Fig. 3D). This showed the advantage of double-strand structure in mechanical property.

XRD was performed on DCDS and catgut suture to compare the crystal size and crystallinity (Fig. 3E). Full width half-maximum (FWHM) can be measured, in terms of 2θ, at an intensity equal to half maximum intensity. Based on formula FWHM = K λ / ε cos θ [51], crystal size (ε) is inversely proportional to FWHM. With smaller FWHM (Fig. 3f1), crystal size of DCDS was larger and the degradation was slower than catgut [52], explaining why DCDS degraded slower than catgut suture. Crystallinity of DCDS suture was calculated higher than catgut (Fig. 3f2). As crystallinity rises, stiffness and strength also increase [53], consistent with the result of mechanical property in Fig. 2. In addition, higher crystallinity can better resist acidic or enzymatic corrosion [52]. Therefore, with stronger acid resistance and slower degradation, DCDS suture could potentially maintain better strength in the body.

### Cytotoxicity, degradation and histocompatibility of sutures

**Fig. 4:**
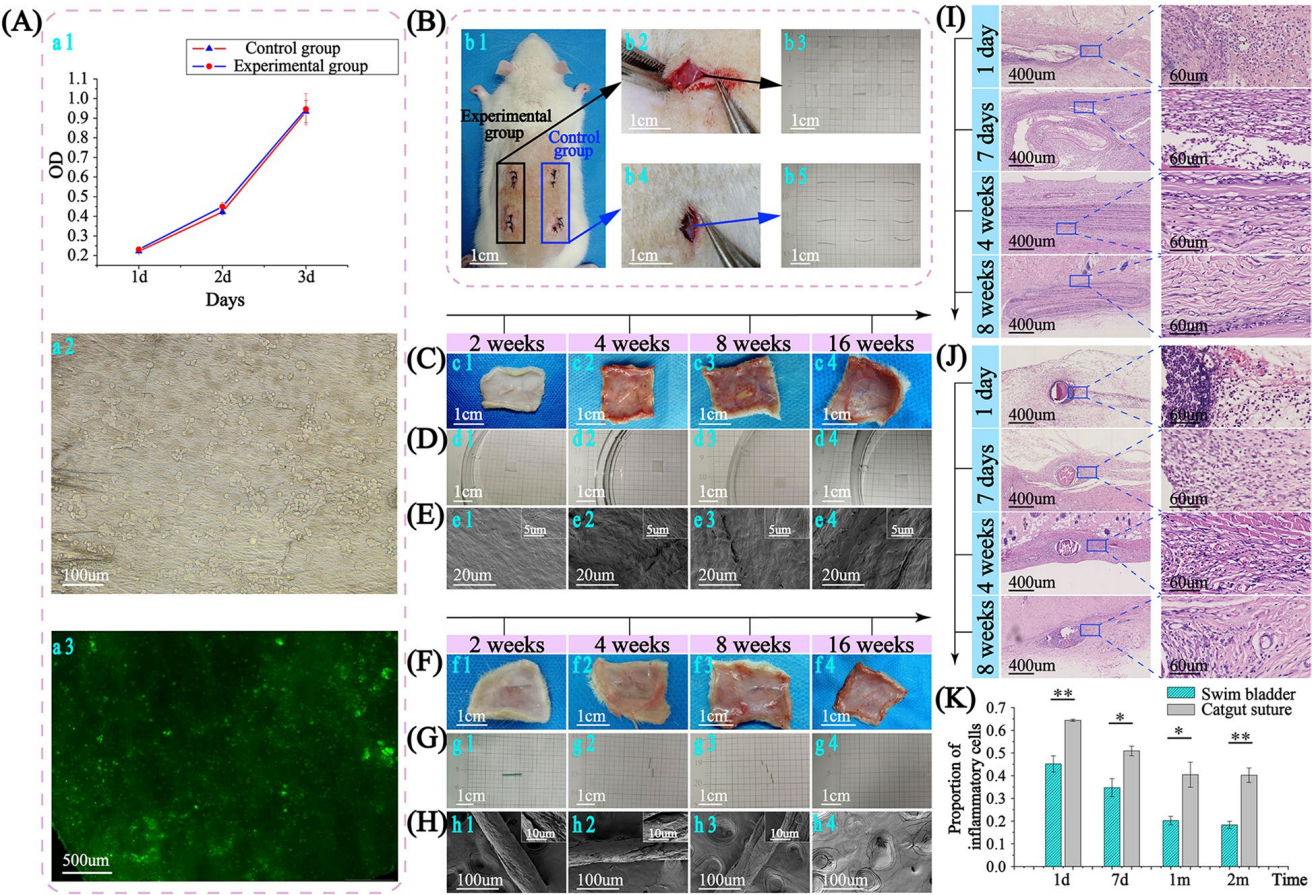
Cytotoxicity, degradation and histocompatibility of sutures. **A** 3T3 growth on the suture and no suture group within 72 hours (*n* = 3). After 1, 2 and 3 days in the experimental group (medium soaked with DCDS suture) and the control group (pure medium) (a1), and 3T3 cells grew on the IDCM (a2, 3). **B** IDCM and catgut suture subcutaneous implantation in rats(b1). The experimental group for IDCM and the control group for catgut sutures. The implantation of IDCM (5 ✕ 5 mm) and catgut sutures (10 ✕ 0.26 mm) shown in b2, b4, and the IDCM and catgut sutures shown in b3, b5. IDCM implanted in tissue samples were visually observed (**C**), on measuring paper (**D**) and observed with SEM (**E**) after 2, 4, 8 and 16 weeks, compared with catgut sutures (**F**-**H**) at the same time points. The HE staining of the rat IDCM (**I**) and catgut sutures (**J**) after 1 day, 1 week, 4 weeks and 8 weeks. At each time point, 4 positions (up, down, left and right) around the implants were selected for inflammatory cells count (**K**) (*n* = 4)

To test cytotoxicity, cells were cultured with pure medium or medium which the IDCM was submerged for 24 hours, and then cell numbers on the different groups were detected. The result showed that the cell numbers of the suture and no suture group both presented a gradual rising trend from the 1st day to the 3rd day (Fig. 4a1). Statistical analysis confirmed that there was no statistical difference between cell numbers of the two groups on the same day, which showing that IDCM was non-cytotoxic and had no inhibitory effect on cell division. IDCM was put into the medium with 3T3 cells for co-cultivation and then were observed with a fluorescence microscope directly (Fig. 4a2) or after staining (Fig. 4a3) to see whether alive cells can be colonized on IDCM. Large numbers of cells can be observed attaching on the surface of IDCM, and can not be washed off by PBS solution, meaning cells can colonize on the surface of IDCM. It indicated that the DCDS suture which was fabricated with IDCM, can be also colonized by cells. After staining, a large number of green cells can be seen under the fluorescence microscope. It showed that most colonized cells were alive, which again proved that the IDCM and DCDS suture were not toxic to cells.

The IDCM (5 ✕ 5 mm), as experimental group, and catgut suture, as control group were implanted subcutaneously in rats (Fig. 4B). The rats were dissected to obtain tissue samples with implants after 2, 4, 8 and 16 weeks. IDCM wrapped in tissue samples can be observed clearly at different weeks (Fig. 4C), meaning that IDCM at different time points were not completely degraded. Then IDCM were taken out from tissue samples and measured with the measuring paper (Fig. 4D). It was found that the size of the IDCM did not change, and there was no visible damage on the surface. Observed under SEM (Fig. 4E), the overall surface of IDCM was smooth, and some cord-like fibers were clear after 2 weeks (Fig. 4e1). After 4 weeks, more prominent cord-like fibers were observed on the surface, and small pores appeared between the fibers (Fig. 4e2). After 8 weeks, prominent cord-like fibers and small pores were more obvious (Fig. 4e3). After 16 weeks, it can be clearly observed that the surface of IDCM was uneven and had obvious hollows, but the overall integrity was still maintained (Fig. 4e4).

The catgut sutures wrapped in tissue samples can be observed after 2, 4 and 8 weeks, but disappeared after 16 weeks (Fig. 4F). Then catgut sutures at different weeks were taken out and measured with measuring paper (Fig. 4G). Catgut suture maintained good integrity after 2 weeks, was easily broken into 2 pieces when picked up after 4 weeks, and was more fragile and easily broken into 3–4 pieces after 8 weeks. After 16 weeks, the catgut suture was not found, indicating that it was completely degraded. Finally, catgut sutures were sent for observation under SEM (Fig. 4H). Catgut’s suture was slightly damaged, and the whole suture remained cylindrical after 2 weeks. Then some deep cracks appeared after 4 weeks, and the integrity gradually deteriorated. After 8 weeks, besides cracks, there were also large missing pieces, and the integrity of the suture cannot be maintained, and then, after 16 weeks, catgut suture cannot be found. Although the IDCM gradually degrade during the 16 weeks after the operation, the overall integrity is not destroyed, and it is not easy to break when picked up. In contrast, the integrity of the catgut sutures is gradually deteriorated and more fragile as time goes on until completely degraded by 16 weeks. It shows that the IDCM can be gradually degraded, while the degradation rate is slower than catgut suture. This can be explained by the crystal size. In Fig. 3, The crystal size of IDCM is larger than catgut. The larger the grain size, the slower the degradation. In addition, higher crystallinity and more thorough cross-linking, which leads to tight bonding between fibers all affect the rate of degradation. The integrity of IDCM can be maintained more than 16 weeks, so, as a suture, DCDS, which is fabricated with IDCM, can more continuously maintain stable tension on the wound and prevent the wound from splitting.

Rats were dissected to obtain the tissue samples with implants after 1 day, 7 days, 4 weeks and 8 weeks, and then samples were sent for pathological sections with HE staining. In the image of HE staining, the inflammatory cells around the IDCM distributed evenly and sparsely after 1 day (Fig. 4I). However, inflammatory cells were very densely distributed around the catgut sutures after 1 day and formed an area of black clumps, showing more severe inflammation (Fig. 4J). Then inflammatory cells in both groups began to decrease gradually. However, the number of inflammatory cells around IDCM was still significantly less than that of catgut sutures at the same time point. At each time point, 4 positions (up, down, left and right) around the implant were selected for inflammatory cell count and calculation of inflammatory cells / nucleated cells under the same magnification (Fig. 4K). The proportion of inflammatory cells around the IDCM is significantly less than the catgut suture at each time point, indicating that IDCM and DCDS suture has better histocompatibility and less inflammation than the catgut suture.

### Acid resistance of sutures

When sutures are used to seal gastric fistulas, strong resistance to acid are required due to extremely low pH in the stomach. Stronger resistance to acid can better avoid corrosion and break of suture caused by gastric acid, and can reduce occurrence of peritonitis caused by that stomach content flows into abdominal cavity. A suture with strong acid resistance, undoubtedly can play a better role in gastric suture surgery.

**Fig. 5:**
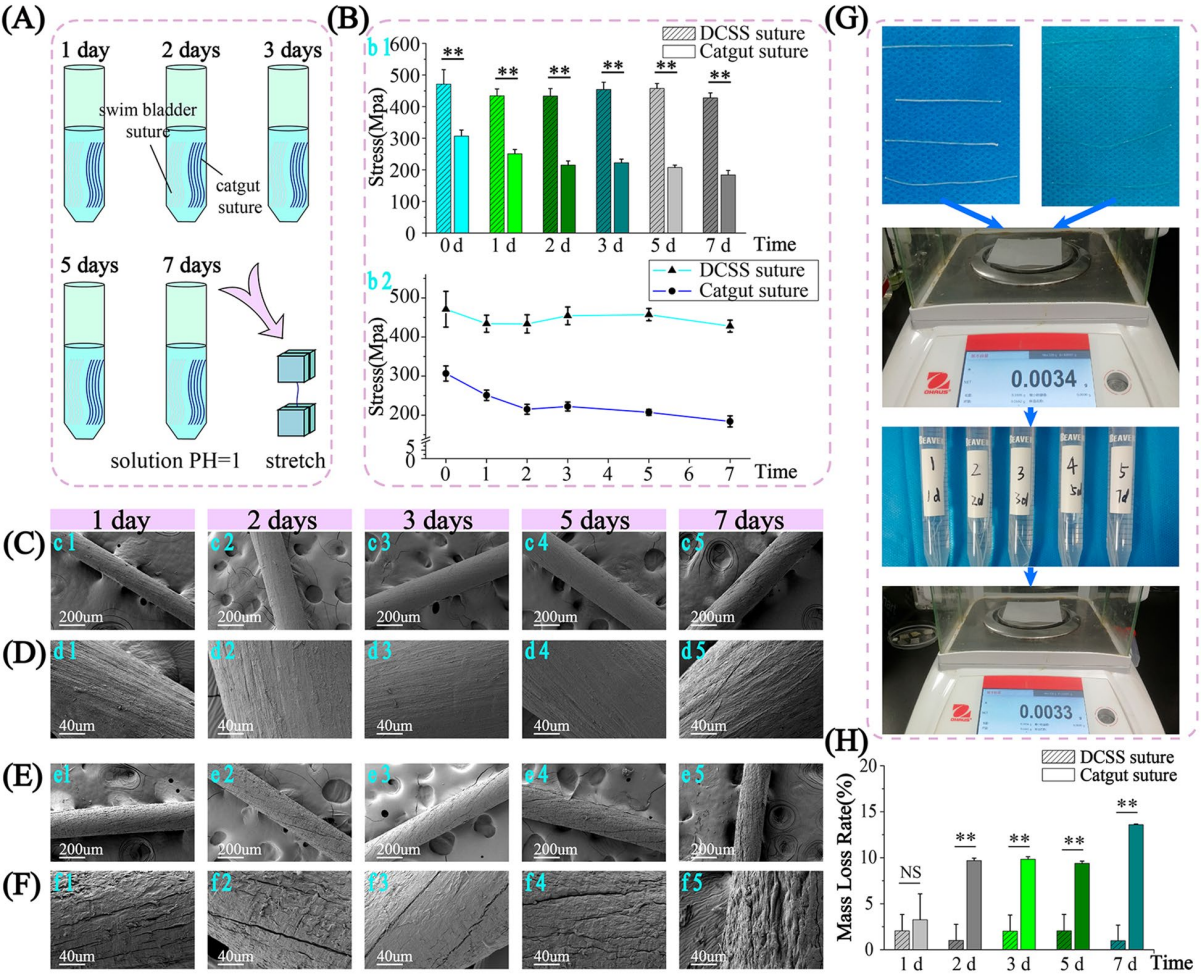
Acid resistance experiment under simulated stomach environment. In the acid solution of pH = 1 (**A**), the tensile strength change with the time course of the sutures of DCSS and catgut (**B**) (*n* = 5). The SEM morphology investigation of DCSS (**C**) (**D**) and catgut sutures (**E**) (**F**). **G** After soaking in acid solution of pH = 1 and weighing, the mass loss rate change with the time course of the sutures of DCSS and catgut (*n* = 3) (**H**)

The DCSS and catgut sutures were soaked in PBS solution of pH = 1 for 0, 1, 2, 3, 5 and 7 days respectively, and then the tensile strength was tested (Fig. 5A). The tensile strength of DCSS suture shows no statistical difference among different time points (Fig. 5B). While the tensile strength of catgut suture among groups of 0, 1, 2–5 and 7 days shows a statistical difference, meaning tensile strength decreases gradually as time goes on. It indicates that the strength of DCSS suture is much less affected by corrosion of gastric acid than catgut suture within the first week.

Then two sutures soaked in acid solution for different days were observed under SEM (Fig. 5C-F). At low magnification, the surface of all the DCSS sutures soaked for different days were still smooth, and no obvious cracks or defects were observed (Fig. 5C). At high magnification, it can be observed that fiber filaments were tightly arranged, and no cracks or corrosion marks (Fig. 5D). At low magnification, surface of catgut suture soaked for 1 day was relatively smooth (Fig. 5e1), but a few small cracks were observed after zooming in (Fig. 5f1). Cracks can be observed on surface of catgut sutures soaked for 2, 3 and 5 days respectively at low magnification (Fig. 5e2–4), and can be observed more obvious than suture soaked for 1 day (Fig. 5f2–4). Signs of corrosion began to appear on the surface of the suture after soaking for 7 days (Fig. 5e5), and after zooming in, it was clear observed the surface was pitted, the fiber bundles were exposed and broken, and the integrity of the suture was obviously damaged (Fig. 5f5). As the soaking time increases, the surfaces of the DCSS sutures almost do not change, while the corrosion of catgut sutures is getting more and more serious, explaining the result of tensile strength of two sutures.

After soaking in acid solution (pH = 1.0) for different days, the mass loss rate of DCDS and catgut sutures were measured (Fig. 5G) and calculated (Fig. 5H). As soaking time increased, the mass loss rate of DCDS suture did not show a significant increase and always maintained a low level. However, the mass loss rate of catgut suture was gradually increasing over time, showing that longer soaking time in acid was, more serious the integrity damage of catgut suture was. Except for soaking for 1 day, at 2–7 days, the mass loss rate of catgut suture soaking for 2–7 days is all higher than DCDS, with statistical difference. This again verifies the strong resistance to acid of DCDS suture, which is much stronger than catgut suture.

### Multi-functional suture inhibiting the secretion of gastric acid and intragastric pH monitoring

**Fig. 6:**
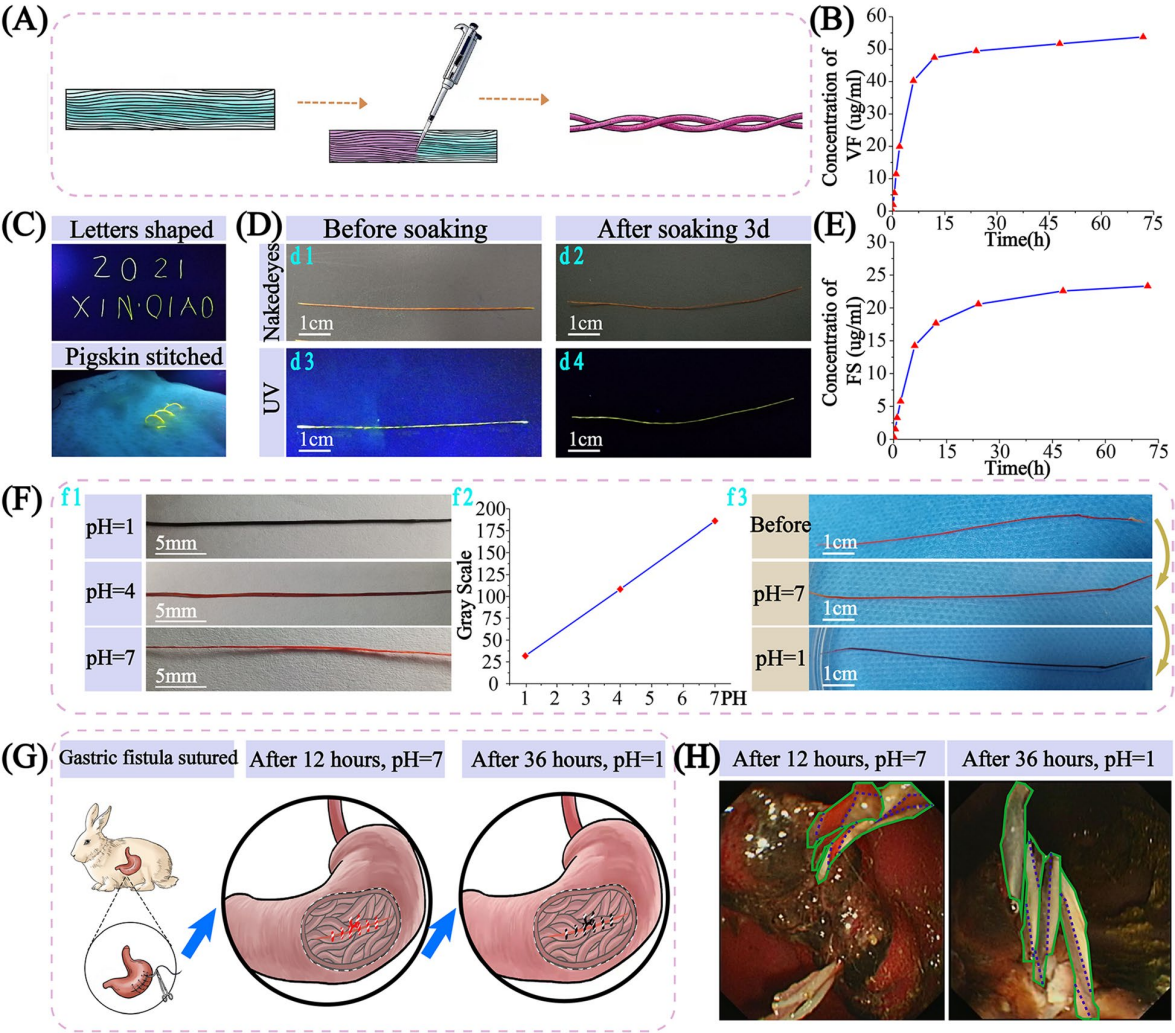
Multi-functional suture for inhibiting gastric acid secretion, fluorescence localization and intragastric pH monitoring. **A** VF, fluorescein sodium and Congo red laden in DCDS suture. **B** Release of VF. **C** DCDS suture with fluorescein sodium (c1) and on the pigskin (c2). **D** Color change of DCDS sutures before (d1) and after soaking in PBS for 3 days (d2) and under UV (d3–4). **E** Fluorescein release curve (FS: Fluorescein sodium). **F** Appearing color (f1) and grayscale (f2) of DCDS sutures with Congo red immersed at different pH solutions, and color was reversible in response to pH change between pH = 1 and 7 (f3). Suturing gastric fistula in rabbits with Congo red and VF (**G**), and color comparison with gastroscopy after the different times (**H**)

DCDS sutures can carry drugs (Fig. 6A) to inhibit gastric acid secretion, position suture, monitor pH of the wound environment. VF is a pyrrole derivative to increase pH in stomach by inhibiting H^+^/K^+^ ATPase. Its cumulative concentration with the time showed a gradual upward with the decreasing release rate (Fig. 6B). After 72 hours, the cumulative curve kept stable, indicating that the suture can maintain a longer-lasting controlled-release effect. DCDS suture with fluorescein sodium emitted yellow-green fluorescence under UV (Fig. 6C). When stitching on pigskin, it can be distinguished with surrounding skin (Fig. 6c2). It can locate the suture after stitching to determine whether the suture was fallen off. The fluorescence intensity of the suture became lighter after soaking for 3 days (Fig. 6D and E).

Congo red loaded suture indicated pH change. Sutures showed blue-black when immersed in pH = 1, dark red in pH = 4 and light red-orange in pH = 7 (Fig. 6f1) with different color intensity in grayscale (Fig. 6f2). The reversible response suggested the suture’s dynamic pH monitoring (Fig. 6f3). DCSS sutures with Congo red and VF were twisted into DCDS to regulate gastric acid secretion and monitor pH. After it was stitched in rabbit stomach, the suture color was close to light red-orange when VF took effect, and then blue-black after VF gradually wore off (Fig. 6G). Same as in the endoscopy, suture color was light red after 12 hours and black after 36 hours (Fig. 6H), indicating pH in stomach was 5–7 after 12 hours and 1–3 after 36 hours. It suggested that the suture inhibited gastric acid secretion. After gastric fistula surgery, the wound will be affected by gastric acid with a risk of bleeding, especially in the first several days. This work provides a new strategy: VF in suture inhibits gastric acid secretion and Congo red dynamically monitors pH in stomach.

### Wound healing of the gastric perforation

**Fig. 7:**
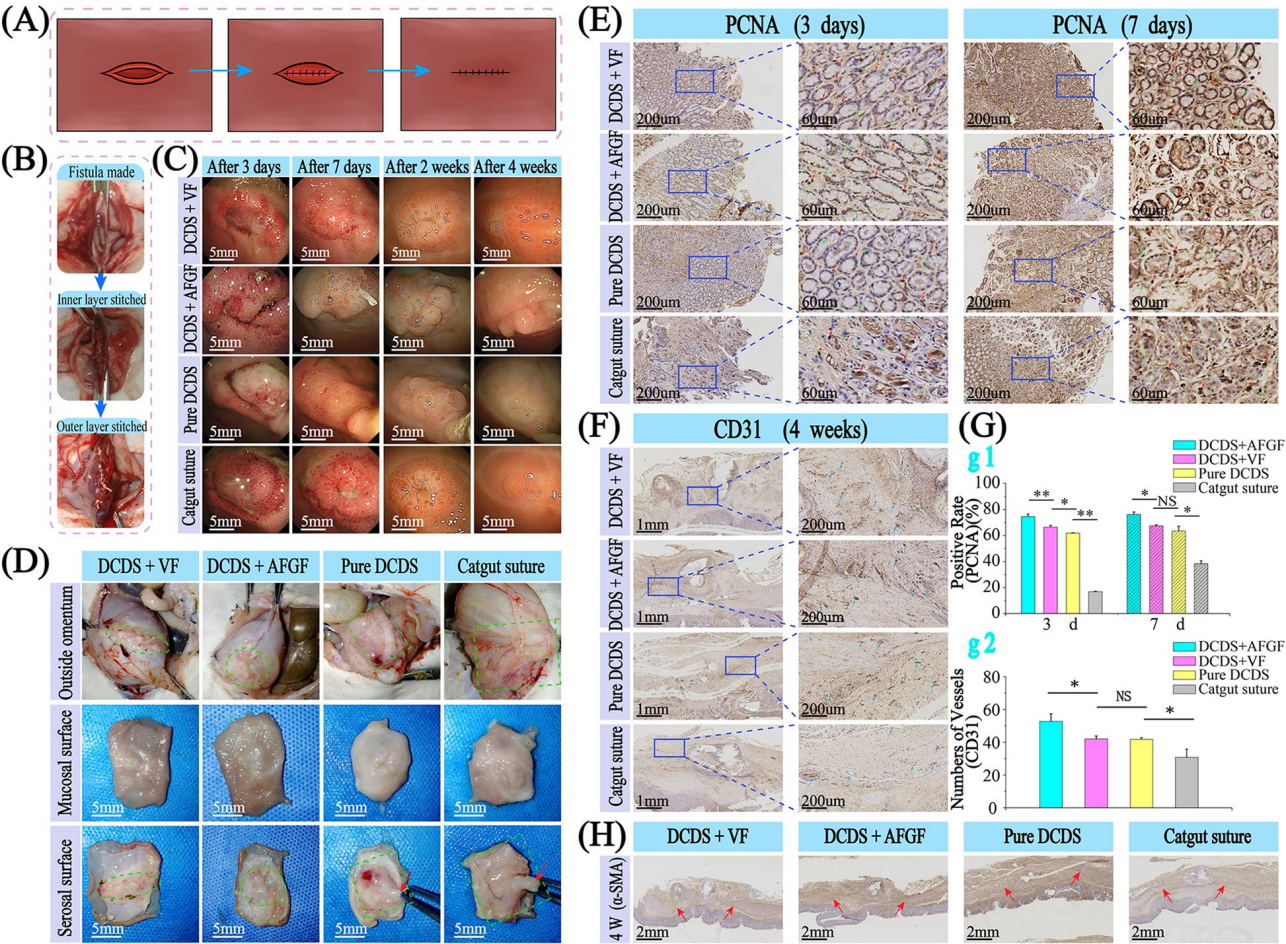
In vivo wound healing. The gastric fistula of rabbit was firstly sutured in the mucosal layer and then in the serosal layer (**A**: schematic diagram, **B**: operation diagram (*n* = 3 in each group)). **C** Endoscopy performed to observe wound healing after 3 days, 7 days, 2 weeks and 4 weeks, respectively. **D** Anatomy and specimen images after 4 weeks. Immunohistochemical staining of PCNA (**E**), CD31 (**F**), and their corresponding histogram graph (*n* = 3) (**G**) and α-SMA (**H**)

After the stomach of the rabbit was stitched with sutures (Fig. 7A, B and Videos 2, 3), endoscopy was performed to observe wound healing (Fig. 7C). After 3 days, redness and swelling showed in all groups, but more severe in DCDS suture with no drugs and catgut group. In the catgut group, obvious purulent exudation was found, which was absent in other groups. After 7 days, the wound healed well with no ulcer in all groups of DCDS sutures (VF, AFGF and no drug groups). Noticeable cell proliferation was seen in the AFGF group, showing the ability to promote healing.

In contrast, a noticeable ulcer was observed in the catgut group, indicating that the wound in the catgut group healed slower than in other groups. It suggested that catgut was worse than DCDS suture in promoting wound healing. Four groups showed no noticeable difference after 2 and 4 weeks. VF group showed an excellent healing effect which may be due to inhibiting gastric acid secretion and thus reducing stimulation on the wound. DCDS suture with no drugs showed a little worse healing effect than the VF and AFGF group but was much better than catgut. After 4 weeks, the outside omentum of the stomach can be observed in all groups, suggesting inflammatory irritation of sutures (Fig. 7D). Only 25–35% area was found in the VF and AFGF groups (Green frame); however, a large area of about 50% in no drug group, and more than 80% in catgut group. Usually, the omentum covers and wraps the tissue at the site of inflammation. Heavier the inflammation irritation is, the more pronounced the adhesion and wrapping of the omentum shows. With a larger and thicker omentum on the surface, the catgut group showed severer inflammatory irritation than DCDS suture groups. PCNA was closely related to cell DNA synthesis. It was a good indicator for wound healing. From immunohistochemical staining of PCNA, brown nuclei (red arrow) present positive for cell proliferation state and blue nuclei (green arrow) present negative (Fig. 7E). After 3 days, brown nuclei were much more than blue in the AFGF group. However, the blue and brown alternate arrangement of nuclei were found in the VF group and the group with no drugs. The least brown nuclei were found in the catgut group. After 7 days, it showed a similar result. The above results showed DCDS suture possessed a better healing effect than catgut, and the effect significantly increased after loading AFGF and VF. Most CD31 staining vessels presented in the AFGF group (blue arrow), then in the VF and no drug groups, and the catgut suture showed the least (Fig. 7F and G). DCDS suture indicated an obvious advantage over catgut. α-SMA staining showed mucosal layer near the wound completely healed, and the muscle layer remained ruptured, confirming the site to obtain tissue was accurate (Fig. 7H).

### In-situ pH monitoring in stomach working with “capsule robot”

**Fig. 8:**
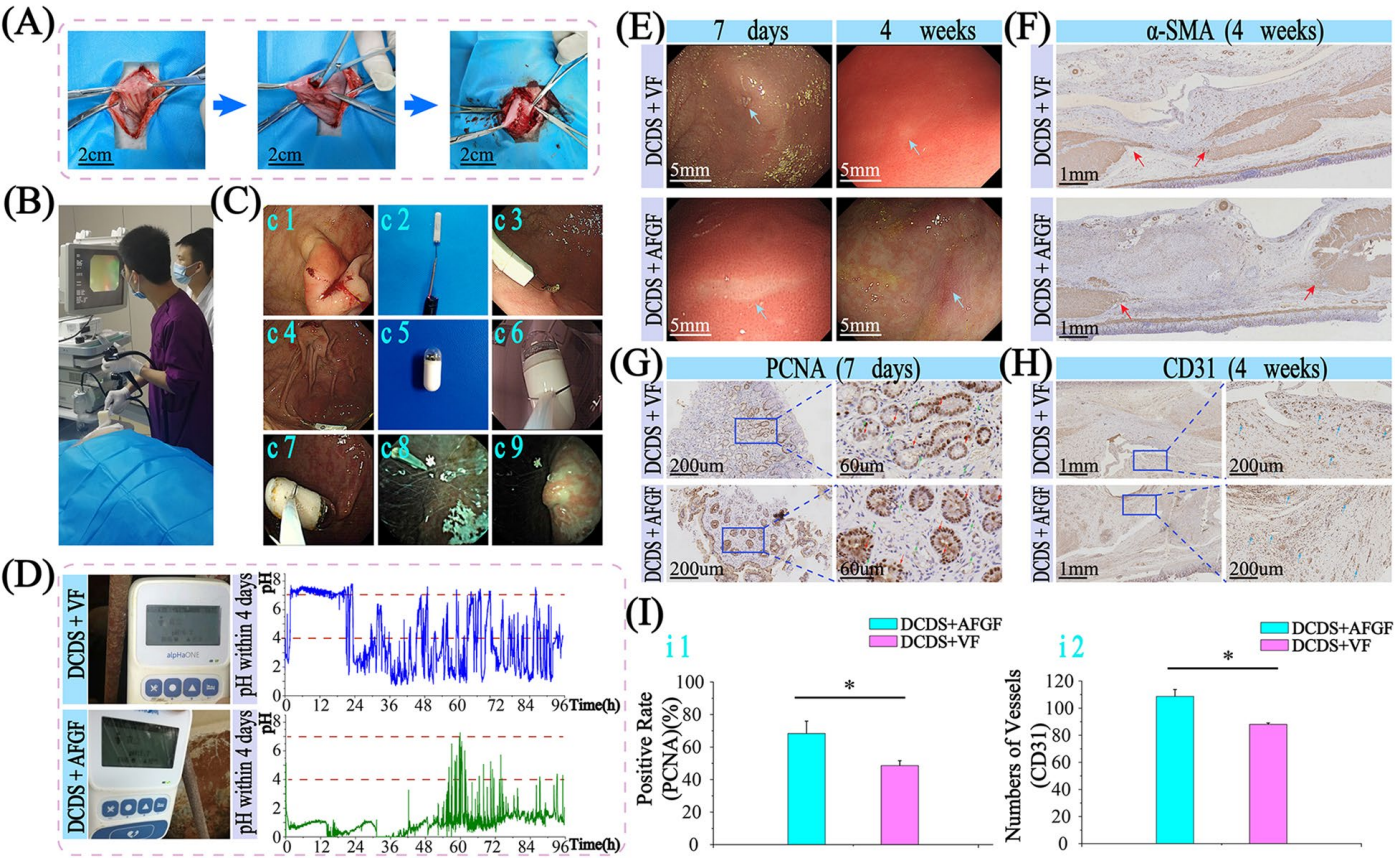
In vivo in-situ real-time “capsule robot” diagnosis and wireless pH monitoring. **A** Gastric fistula of minipig. **B** Gastroscopy operation. General endoscopy, real-time pH wireless meter and “capsule robot” shown in **C** (c1: wound observed from stomach inside, c2: implantable real-time pH wireless meter, c3: pH wireless device in the stomach, c4: pH device near the wound, c5: “capsule robot”, c6: “capsule robot” moved to the stomach, c7: “capsule robot” positioned in the stomach, c8: pH meter taken by “capsule robot” after 7 days, c9: wound morphology monitored and taken by “capsule robot” after 7 days). **D** In-situ pH monitoring. **E** Observation under endoscopy after 7 days and 4 weeks of the AFGF and VF groups. α-SMA (**F**), PCNA (**G**), CD31 (**H**) staining and corresponding histogram graph (*n* = 4) (**I**)

After stitching gastric fistula with DCDS sutures (Fig. 8A, Video 4), implantable pH wireless meter (Video 5) and “capsule robot” (Video 6) were used to study sutures and their effects on the gastric wound (Fig. 8B, C). pH changes in the stomach for the first 4 days was obtained (Fig. 8D). In the VF group, pH was maintained at about 7 during the first 24 hours, compared with a strong acidic environment of pH of 0–1 in the AFGF group. It suggested that DCDS suture with VF inhibited gastric acid secretion and maintained pH in the stomach at 7 for about 24 hours, significantly reducing the occurrence of wound bleeding. After 24 hours, it still can maintain pH > 4 for more than 37 hours in 72 hours, effectively preventing bleeding. In the AFGF group, pH was at 0–1 most time.

After 7 days and 4 weeks, wound healing was investigated using endoscopy and immunohistochemistry staining (Fig. 8E). α-SMA staining showed that mucosal layers were healed entirely, and muscle layers (red arrow) were still both ruptured, confirming the site to obtain tissue was accurate (Fig. 8F). PCNA staining showed that the proportion of brown nuclei in the AFGF group was significantly higher than VF (Fig. 8G, i1). CD31 staining also showed vessels in the AFGF group were more (Fig. 8H, i2). It suggested that DCDS suture with AFGF promoted wound healing better than VF.

### Achilles tendon suture and healing

**Fig. 9:**
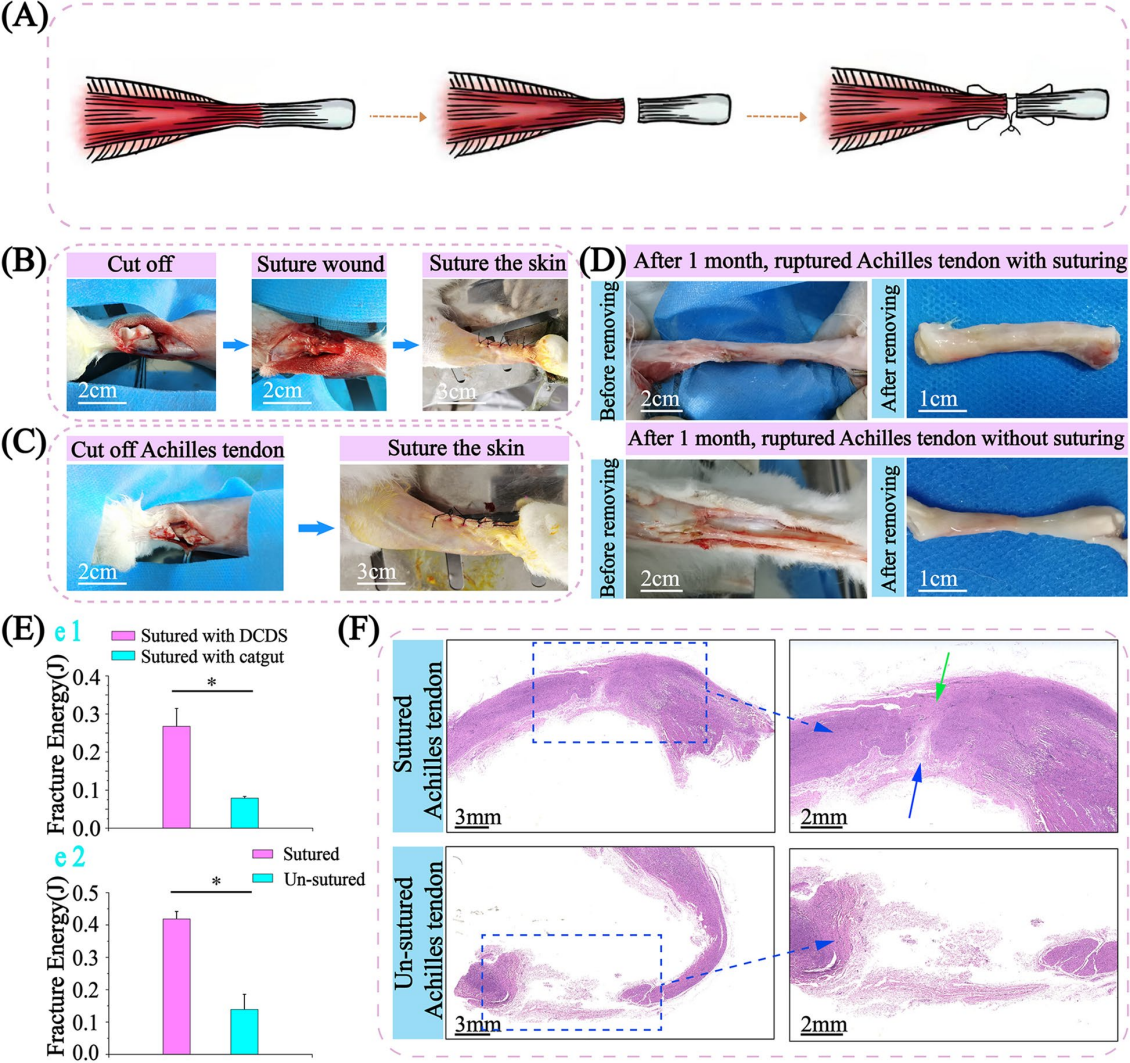
DCDS suture repairing the cut Achilles tendon of rabbit. **A** Suturing illustration of Achilles tendon. DCDS suture stitching the breaking interfaces (**B**), and blank with no treatment as a control group (**C**). **D** Observation of Achilles tendon after 1 month. **E** Fracture energy of sutured Achilles tendon with DCDS and catgut after 1 day (e1), and sutured and un-sutured Achilles tendon after 1 month (e2) (*n* = 3). **F** HE staining of sutured and un-sutured Achilles tendon after 1 month

Achilles tendon bears extremely strong tension [54]. For ruptured tendons, a higher requirement for sutures in strength is needed. A further test was verified for the tough DCDS suture (Fig. 9A-C, Video 7). After 1 month (Fig. 9D), the Achilles tendon in the control group was thinner with fibroblasts spreading and wrapping. Achilles tendon in the DCDS groups was significantly thicker due to the suture’s resistance to maintain anastomosis. The tendon showed uniform thickness from middle (operation site) to ends, while obviously thinner in middle than ends in control group. By stretching (Fig. 9E), fracture energy of DCDS group after 1 day was more than 3 folds higher than catgut group (Fig. 9e1), showing stronger strength of DCDS suture than catgut. After 1 month, fracture energy of the DCDS group was also more than 3 folds higher than the un-sutured group (Fig. 9e2). In HE staining (Fig. 9F), compared with a large amount of scattered mucosa and interrupted tissue in the un-sutured Achilles tendon, interrupted tissue in sutured tendon maintained anastomosed and was in the healing process. One part of the tendon was connected with proliferated fibrous tissue (green arrow), showing good wound healing. The other part (blue arrow) showed it was still under the healing process. DCDS sutures played a significant role in promoting healing and maintaining connection status between broken ends. This was also verified in the movement state of rabbits after 1 week and 2 weeks. Rabbits sutured with DCDS moved faster than un-sutured (Video 8).

## Discussion

Overall characteristics of SB tested in this work show it is suitable as a new absorbable suture. SB is non-toxic, and it can be colonized by cells (Fig. 4), showing cells can proliferate on surface of DCDS suture to get a better interaction between suture and tissue. After crosslinking, with significantly higher wet strength and rigidity than catgut (Fig. 2), DCDS suture can be better applied in body fluids environment and maintain complete shape and wound fitting without cracking, avoiding sutures or wound breaking. This is also verified in Achilles tendon experiments (Fig. 9). As one of the most tensile-bearing tendons in the body, it proposes extremely high requirements in suture strength. DCDS suture successfully repairs the ruptured Achilles tendon and shows excellent strength.

Gastric perforation may cause severe peritonitis even death after stomach contents flow into the abdominal cavity [55, 56]. Although novel preventive and therapeutic measures are being explored [57, 58], the usual treatment is still stitching fistula, requiring better suture acid resistance to low pH in the stomach. Catgut, one of the commonly used sutures in the surgery, is verified rapid strength loss in simulated gastric acid (Fig. 5). The stomach wall is repetitively peristaltic. The strength of the catgut suture begins to decrease after soaking in the acid solution for 1 day, increasing the possibility of wound dehiscence. DCDS suture possesses stronger acid resistance and slower degradation. No obvious corrosion signs and statistical difference in strength exist even after soaking in the acid solution for 7 days (Fig. 5). With stronger acid resistance and higher wet strength, DCDS suture can better avoid damage caused by acid, showing greater advantages than catgut. In addition, the properties of DCDS suture are affected by crosslinking density, which can be regulated by the concentration of crosslinking agent and time [59, 60]. In the clinic, different surgeries for organs and tissues propose different requirements for sutures. According to different needs, sutures with different crosslinking density can be selected. This is also the content of our next study.

Micro-pores exist between fibers, allowing air to pass through [61]. It is a great innovation to allow drugs to pass through the membrane, achieving slow releasing (Fig. 6). Traditionally, drug coated on suture surface is difficult to take effect for long time [62, 63]. In some environments, drugs even may become inactivated. Sutures with several drugs on the surface cannot release at the same time, usually from outer to inner. Although drugs in the inner may release after heated, it is complicated and uncontrollable [2]. In addition, drug coating is usually fragile and easily damaged, which is a significant limitation. In our work, the drug on the membrane surface is rolled in the suture and released through the membrane. Concentric multi-layer structure in cross-section causes different release speed, faster in outer and slower in inner, achieving sustained releasing. Although the drug effect gradually decreases, it can still be maintained as drugs diffuse from inside to outside. Drugs can also be protected in the suture, reducing the possibility of inactivation and avoiding drug damage. Double-strand structure can carry two drugs to achieve independent and coordinated efficacy, and it may carry four after both sides of membrane lade drugs, which needs further exploration. With a simple process, diverse functions and multi-drug combination option, DCDS suture shows significant advantage as a multi-functional suture.

pH in the stomach is about 1, affecting wound healing and increasing bleeding possibility. Bleeding will easily occur when pH < 4 [30, 64], and benefits are shown after pH > 6 during bleeding [65–67]. In our work, DCDS suture with VF can maintain pH > 7 for 24 hours, pH > 4 for more than half of measurement time, greatly reducing bleeding risk during first few days. Based on this, DCDS suture with VF and Congo red can increase pH in the stomach with real-time monitoring at the same time. Different from other multi-functional sutures with a single function or separated multi-functions, DCDS suture with drugs can perfectly combine different functions together, opening up a new idea of therapy. A functional combination of inhibiting gastric acid secretion and real-time pH monitoring only provides a basic idea. Further applications of different drugs laden in sutures still need exploration.

The DCDS suture length is limited by the size of the swim bladder, but its length is sufficient for use in clinic. In clinical application, the length of most absorbable sutures is 30–50 cm. Some barbed sutures have around 20 cm. During surgery, long sutures are often needed to be cut into a few sections for use. At present, the longest DCDS suture we make can be as long as almost 20 cm. Usually, the instrument is used to tie the knot during internal organ surgery, and the DCDS suture with a length of 20 cm is long enough for instrument knotting. To perfectly solve the limitation is also a part of our following study.

## Conclusions

Overall characteristics of SB tested in this work show it is suitable as a new absorbable suture. DCDS suture is non-toxic and can be colonized by cells, and shows significantly higher strength than catgut. After carrying drug, it can achieve different combinations of drugs according to different treatment. A functional combination of inhibiting gastric acid secretion and real-time pH monitoring only provides a basic idea. Even it may achieve more functions after both sides of membrane lade drugs. Further applications of different drugs laden in sutures still need exploration.

## Supplementary Information

**Additional file 1: Supplementary Fig. S1.** PGA with highly complex structure under SEM. **Supplementary Fig. S2.** Two other crosslinking methods. **Supplementary Fig. S3.** The detailed process of fabricating DCDS suture with standardization. **Supplementary Fig. S4.** The tensile strength of double-layers swim bladder with and without crosslinking.

**Additional file 2: Video 1.** Fabrication of DCSS suture without crosslinking.

**Additional file 3: Video 2.** Schematic process of gastric perforation surgery.

**Additional file 4: Video 3.** Operation process of gastric perforation surgery on rabbit.

**Additional file 5: Video 4.** Operation process of gastric perforation surgery on minipig.

**Additional file 6: Video 5.** The process of pH wireless meter sent into stomach.

**Additional file 7: Video 6.** The process of “capsule robot” sent into stomach and observing.

**Additional file 8: Video 7.** Process of suture for Achilles tendon.

**Additional file 9: Video 8.** Comparison of rabbits’ mobility in two groups after 1 and 2 weeks.

## Data Availability

All data associated with the study have not been deposited in a public repository but are available from the lead contact upon reasonable request.

## Abbreviations

PLGA: Polyglycolic-co-lactic acid
SB: Swim bladder
DCDS: Decellularized crosslinked double-strand swim bladder
DCSS: Decellularized crosslinked single-strand swim bladder
DUSS: Decellularized un-crosslinked single-strand swim bladder
VF: Vonoprazan Fumarate
AFGF: Acidic fibroblast growth factor
SDS: Sodium dodecyl sulfate
HE: Hematoxylin-eosin
α-SMA: α-Smooth muscle actin
PCNA: Proliferating cell nuclear antigen
FS: Fluorescein sodium
DCM: Decellularized crosslinked membrane
DUM: Decellularized un-crosslinked membrane
SEM: Scanning electron microscope
FTIR: Fourier transform infrared
IDUM: Inner-layer DUM
IDCM: Inner-layer DCM
SAXS/WAXS: Small/Wide angle X-ray scattering
XRD: X-ray diffraction
UPLC: Ultra-performance liquid chromatography
UV: Ultraviolet
FWHM: Full width half-maximum
