# Supplementary material for: Aligned nanofibrous collagen membranes from fish swim bladder as a tough and acid-resistant suture for pH-regulated stomach perforation and tendon rupture
Source: Biomater Res. 2022 Nov 8;26:60. doi: 10.1186/s40824-022-00306-1 (PMC9641846; doi:10.1186/s40824-022-00306-1)
Supplement: Supplementary file 1 — Additional file 1: Supplementary Fig. S1. PGA with highly complex structure under SEM. Supplementary Fig. S2. Two other crosslinking methods. Supplementary Fig. S3. The detailed process of fabricating DCDS suture with standardization. Supplementary Fig. S4. The tensile strength of double-layers swim bladder with and without crosslinking. [file 40824_2022_306_MOESM1_ESM.doc]

**Supplementary figures**


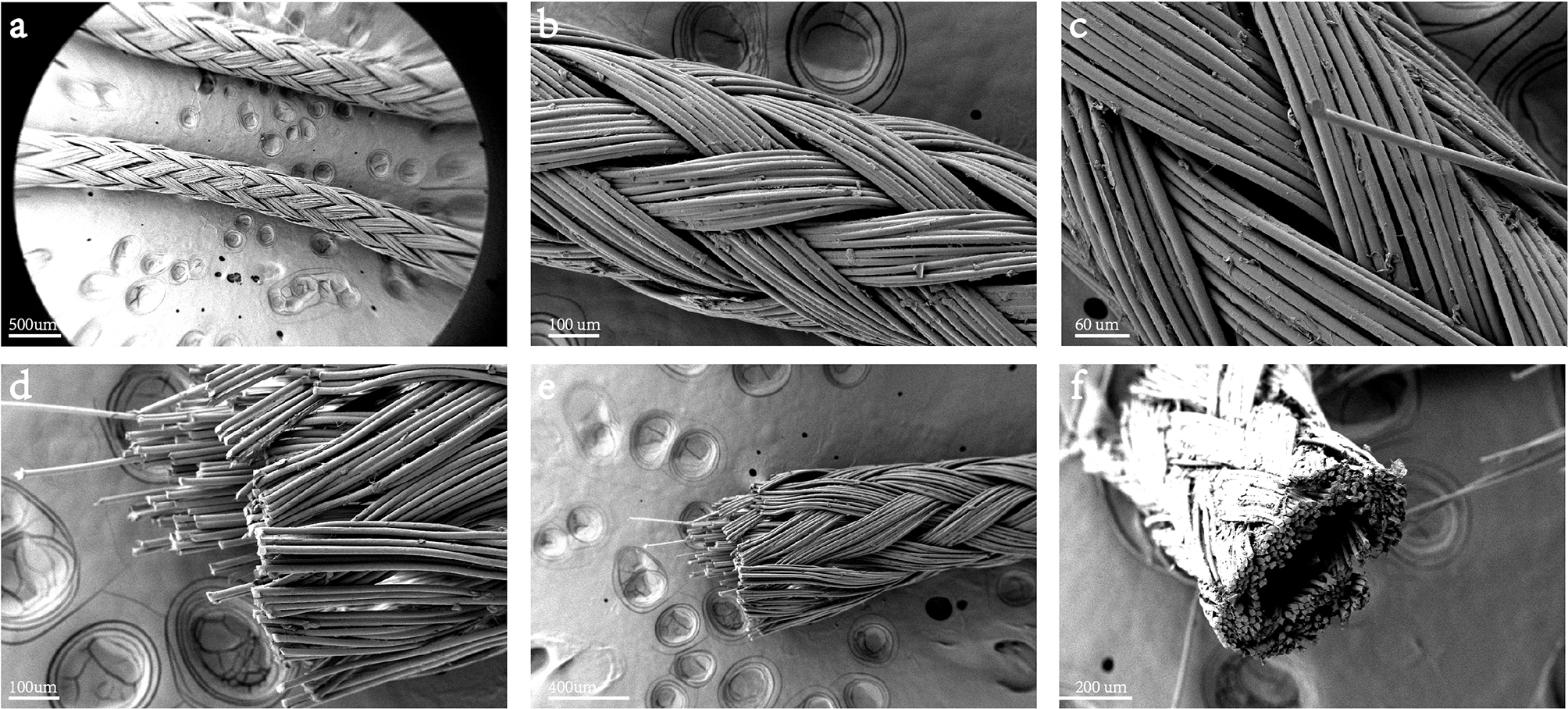


**Supplementary Fig.S1:** PGA with highly complex structure under SEM.


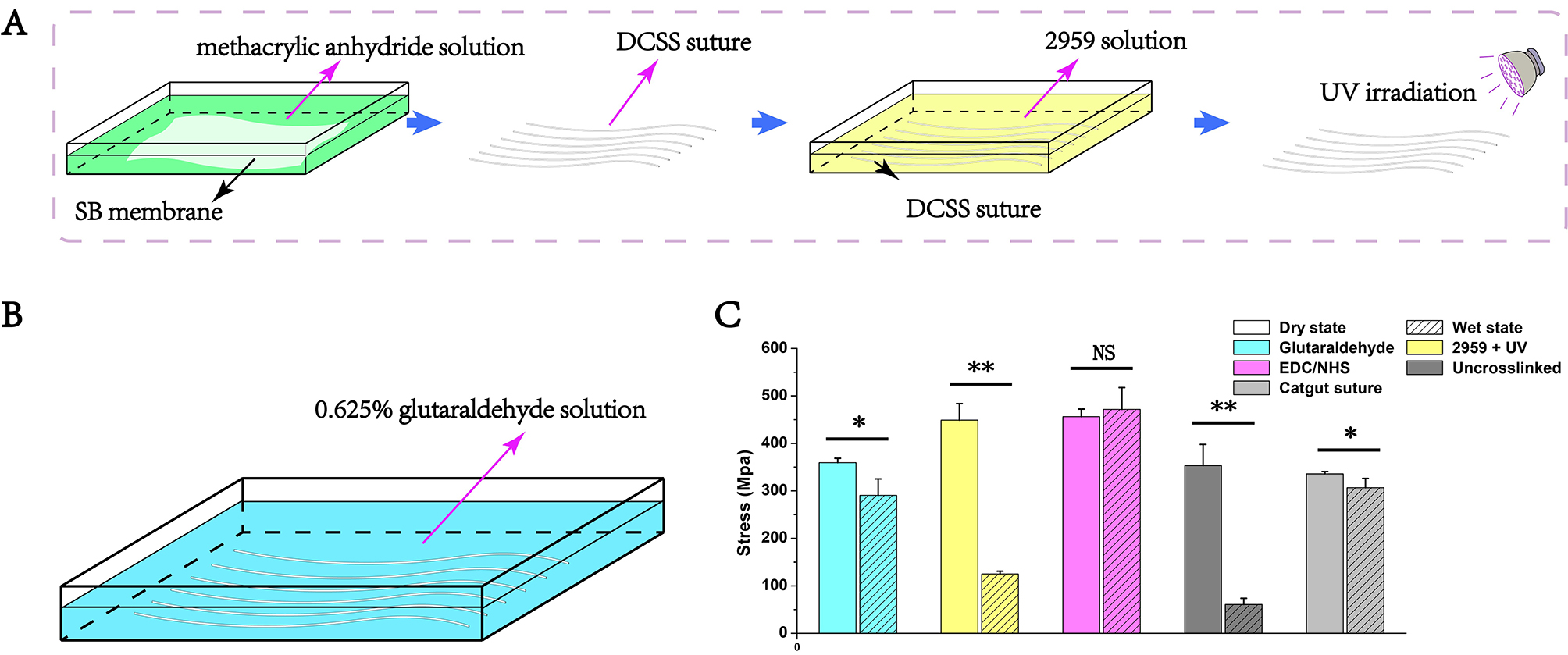


**Supplementary Fig.S2:** Two other crosslinking methods.

(A) IDUM was firstly submerged in methacrylic anhydride solution, and then was fabricated into sutures. Then suture was submerged in 2959 solution and then irradiated under UV for crosslinking. (B) Sutures were submerged in 0.625% glutaraldehyde solution for crosslinking. (C) Tensile strength of different treated DCDS sutures and catgut sutures in the dry and wet states.


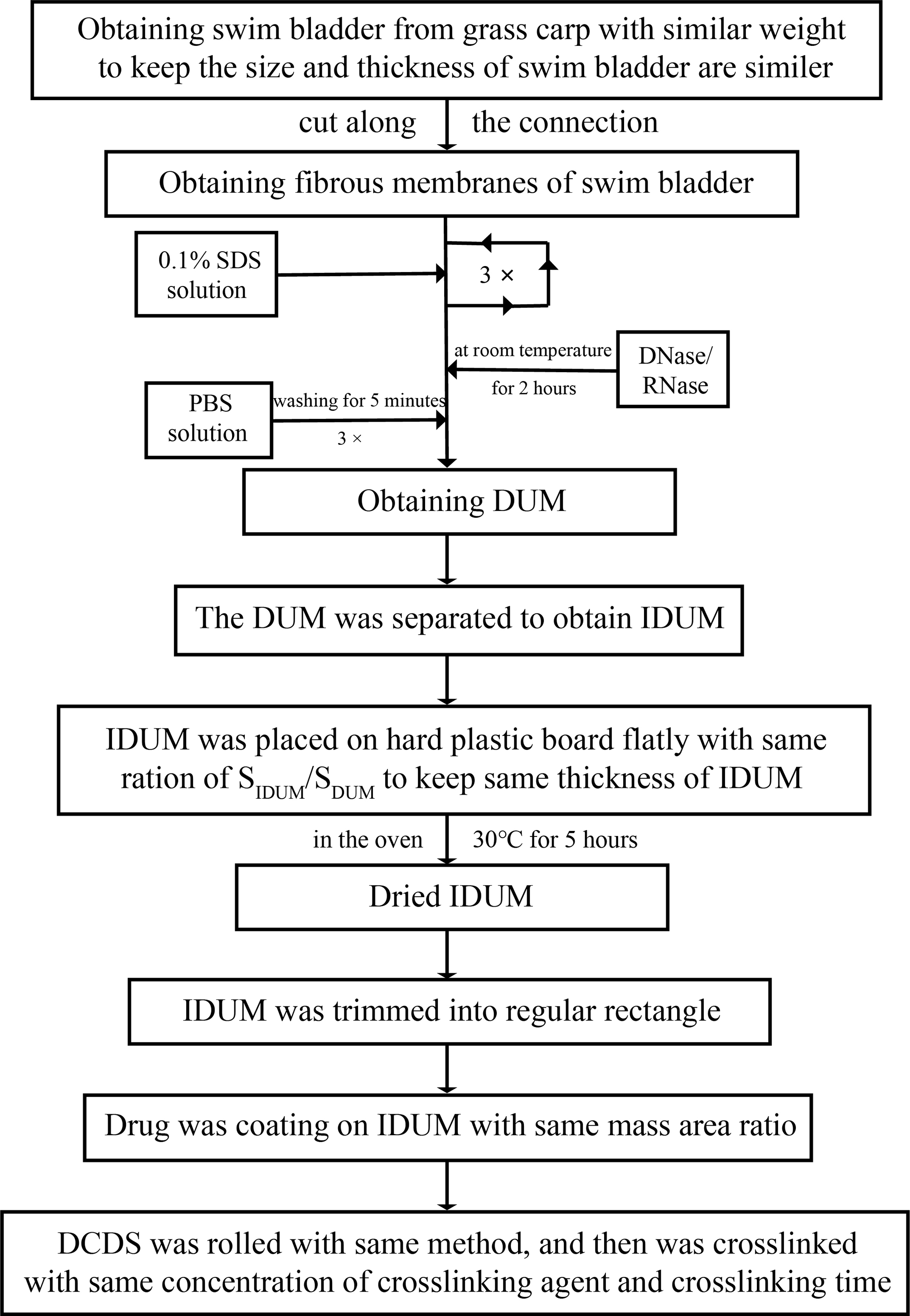


**Supplementary Fig.S3:** The detailed process of fabricating DCDS suture with standardization.


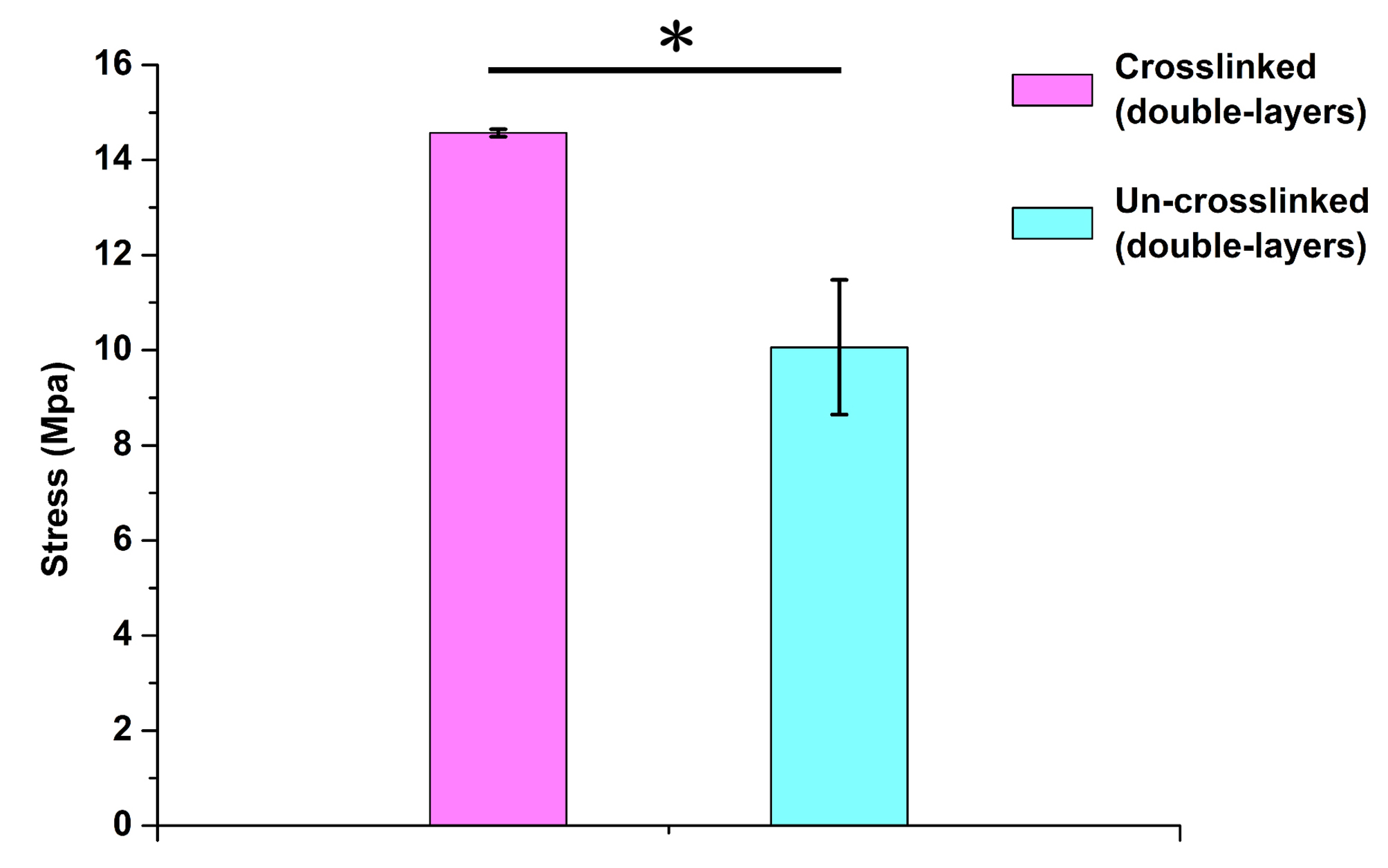


**Supplementary Fig.S4:** The tensile strength of double-layers swim bladder with and without crosslinking.
